# Role of Interleukin-6 Mediated T Cell Activation in Experimental Acute Pancreatitis

**DOI:** 10.1007/s10753-025-02329-x

**Published:** 2025-06-25

**Authors:** Juliane Glaubitz, Anna Zimdahl, Sebastian Zeissig, F. Ulrich Weiss, Matthias Sendler

**Affiliations:** Department of Medicine A, University Medicine Greifswald, Fleischmannstr. 41, 17475 Greifswald, Germany

**Keywords:** Acute pancreatitis, Interleukin-6, T cell activation, Immune regulation

## Abstract

Acute pancreatitis (AP) is an inflammatory disease characterized by a prominent local infiltration and activation of innate immune cells, but also accompanied by a systemic T cell activation. The mechanisms which link the local immune response to the systemic immune activation are not well understood. Here, we identified Interleukin-6 (IL-6) as a crucial mediator of systemic T cell activation, which is mainly released by pancreatic macrophages and necrotic acinar cells. IL-6 triggers the systemic T cell activation of CD4^+^ T helper cells. After the onset of acute pancreatitis IL-6 serum levels increase rapidly and affect the systemic immune reaction. A CD4^+^ specific knock out of the IL-6 receptor glycoprotein 130 receptor (GP130R), showed reduced proliferation and a higher apoptosis rate of T cells after induction of caerulein-induced AP. This diminished T cell activation results in a significant decrease of pancreatic macrophage proliferation and a milder disease course. Our data indicate a critical role of IL-6 in the crosstalk between local and systemic immune responses during AP, as well as a triggering role in adaptive immune mechanisms.

## Introduction

Acute pancreatitis (AP) is one of the most common non-malignant diseases of the gastrointestinal tract, which results in hospitalization of patients, with an increased incidence in recent years [1, 2]. In approximately 80% of cases, AP is characterized by a mild and self-limiting course. However, depending on the disease severity, pancreatitis is life-threatening in 15–20% of cases [3, 4]. Until now, there is no specific drug therapy for AP available and only symptomatic treatment consisting of intravenous fluid and pain relievers is used in clinical routine. These limited therapeutic options are due to the fact that the disease pathomechanism underlying AP are not well understood.

AP has been described as self-digestion of the pancreas by its own enzymes [2]. The intracellular activation of digestive enzymes within pancreatic acinar cells results in necrotic cell death [5] and induces a prominent local immune reaction which also evokes a systemic immune response. An overwhelming inflammatory reaction can ensue with severe complications, such as multi organ failure or infection of pancreatic necrosis [1, 2, 6]. Severity and clinical course of acute pancreatitis are mainly influenced by the immune response [7]. Experimental animal studies have shown that within hours after the induction of pancreatitis, neutrophil granulocytes [8] and especially macrophages migrate and accumulate in the pancreas [9–11]. Necrotic acinar cells release damage associated molecular patterns (DAMPs) such as free DNA, free histones or ATP, which activate macrophages/monocytes via their toll-like receptors (TLR). In response, the cells release cytokines, such as Interleukin-6 (IL-6) [6]. IL-6 is a member of the IL-6 cytokine family and plays a central role in many inflammatory diseases. Other members of the IL-6 family are IL-11, IL-27, IL-35, Oncostatin-M (OSM), leukemia inhibitory factor (LIF), ciliary neurotrophic factor (CNTF), cardiotrophin (CT-1) and cardiotrophin-like cytokine factor 1 (CLCF1) [12]. Cytokines of the IL-6 family are involved in the differentiation, homeostasis and proliferation of lymphocytes and endothelial cells [13, 14] and all family members mediate their stimulating effect via a common β-receptor subunit glycoprotein 130 kDa (GP130), while a specific α-receptor subunit binds the individual cytokine, like in case of the IL-6 receptor (IL-6Rα) which specifically binds the cytokine IL-6 [15]. In the absence of an inflammatory reaction there is no systemic IL-6 detectable, but under inflammatory conditions serum IL-6 concentrations increase up to a thousand-fold [16]. Patients suffering from AP show elevated serum levels of IL-6 and it is assumed that the serum concentration of IL-6 correlates with multi organ failure [17]. The role of IL-6 as early prediction marker of AP is still under debate [17, 18]. In a mouse model of AP, pancreatitis induced hyperinflammation was accompanied by systemic immunosuppression, which is characterized by the induction of CD4^+^ T cells and in particular regulatory T cells [6, 19]. However, it has not been shown how T cells become activated in such a rapid way. It is assumed that the initial local proinflammation, which is characterized by the infiltration of neutrophils and monocytes/macrophages, determines the development of a systemic immunosuppression by the release of cytokines. IL-6 is known to play a crucial role in T cell expansion, function and migration under inflammatory conditions [20, 21]. Here, we investigated in a mouse model of AP the effect of IL-6 and OSM on T cell activation and the local pancreatic damage.

## Results

### Cytokines of the IL-6 family are released during the course of pancreatitis

**Fig. 1:**
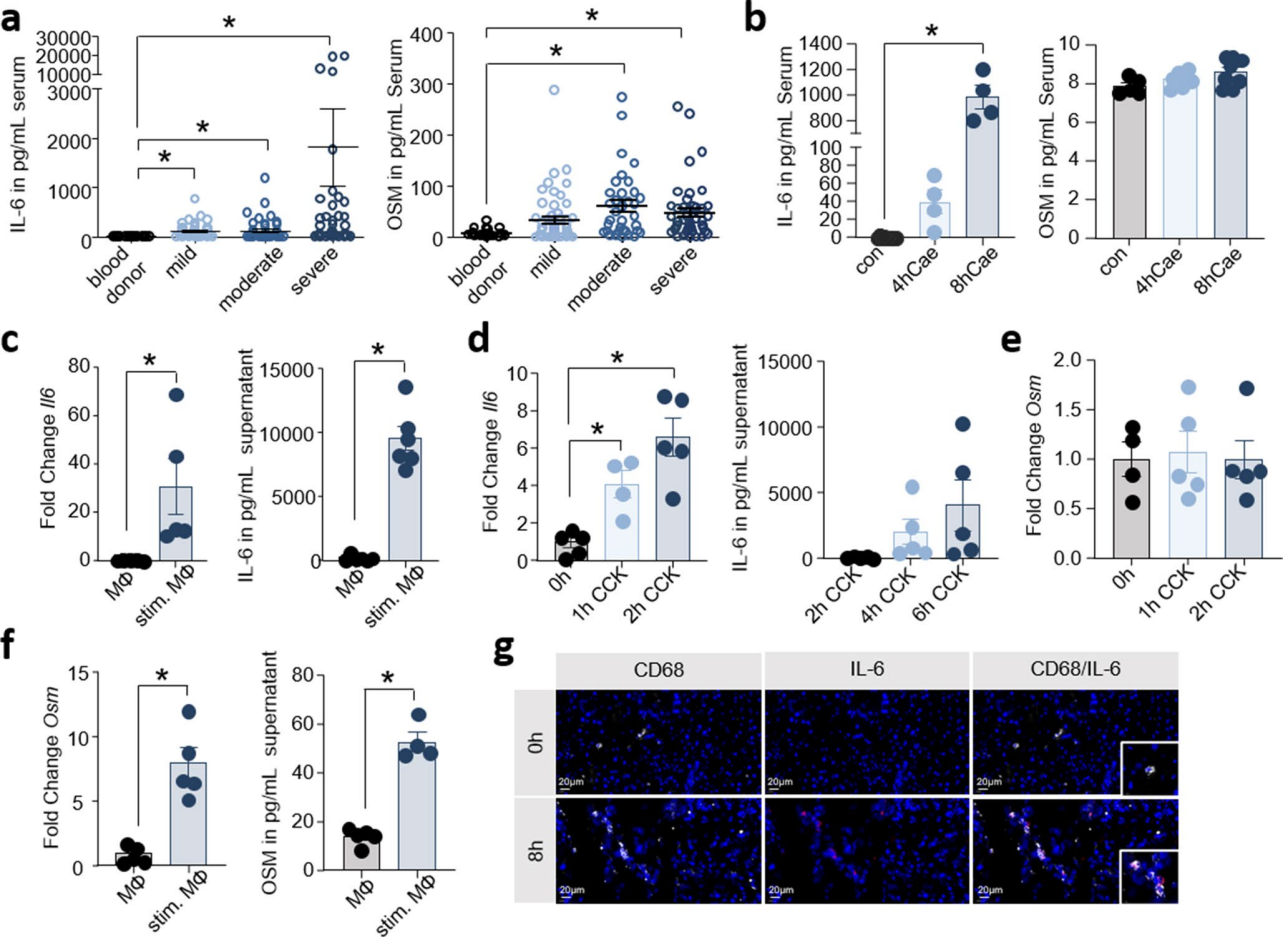
Cytokines of the Interleukin-6 family are released during the course of pancreatitis. (**a**) Serum Interleukin-6 (IL-6) (mild: *n* = 39 mean = 126.93 pg/mL, moderate: *n* = 46 mean = 129.47 pg/mL, severe *n* = 39 mean = 1815.89 pg/mL) and Oncostatin M (OSM) (mild: *n* = 54 mean = 33.45 pg/mL, moderate: *n* = 35 mean = 60.10 pg/mL, severe *n* = 46 mean = 47.36 pg/mL) concentrations were analyzed in acute pancreatitis patients and 20 blood donors (IL-6 mean 2.8 pg/mL, OSM mean 7.85 pg/mL). (**b**) Serum IL-6 and OSM levels were measured 4 h (IL-6 *n* = 4, OSM *n* = 7) and 8 h (IL-6 *n* = 4, OSM *n* = 8) after the onset of experimental pancreatitis in mice (control IL-6 *n* = 10, OSM *n* = 5). (**c**) Gene expression analysis of *Il6* in pancreatic macrophages (MΦ) following stimulation with damaged acinar cells (stim. MΦ) (con *n* = 5, stim. MΦ *n* = 5). Cytokine release of IL-6 was measured in cell supernatant (con *n* = 6, stim. *n* = 6). (**d**) Gene expression changes of *Il6* in freshly isolated acinar cells were analyzed by qRT-PCR under stimulation with 1 µM Cholecystokinin (CCK) (0 h *n* = 5, 1 h *n* = 4, 2 h *n* = 5). Release of IL-6 by acinar cells was determined after 2 h, 4 h and 6 h (2 h *n* = 5, 4 h *n* = 5, 6 h *n* = 5). (**e**) Gene expression of *Osm* in acinar cells (0 h *n* = 4, 1 h *n* = 5, 2 h *n* = 5). (**f**) Gene expression analysis of *Osm* in pancreatic macrophages (con *n* = 6, stim. *n* = 5) and cytokine release of OSM was measured in supernatant from stimulated pancreatic macrophages with damaged acinar cells (con *n* = 6, stim. MΦ *n* = 5). (**g**) Immunofluorescence labeling of IL-6 and CD68 on CD68^+^ macrophages demonstrated a co-localization (Scale bar, 20 µM). All biological replicates (n) are shown +/- SEM and means. Student’s t-test (two-tailed) (**c**,** f**), ANOVA (**d**) or Kruskal-Wallis test (**a**,** b**) was used to determine statistical significance, which was indicated by an asterisk at a *p* value < 0.05

In a first step, we measured serum concentrations of IL-6 and OSM in 124 AP patients, which had retrospectively been classified according to the revised Atlanta classification system. This system categorizes acute pancreatitis (AP) into the following forms: mild (absence of organ failure and local or systemic complications); moderate (transient organ failure lasting < 48 h); and severe (persistent organ failure > 48 h) [22]. Age and sex matched blood donors were used as control group. The serum concentrations of IL-6 as well as OSM were significantly elevated in AP patients in a disease severity-dependent manner (Fig. 1a). Based on this observation, we evaluated serum IL-6 and OSM in a mouse model of acute pancreatitis. AP was induced in C57Bl/6-J mice by hourly injections of caerulein. The serum concentration of IL-6 increased significantly after 8 h while serum levels of OSM were not affected (Fig. 1b). In the next step, we sought to identify the cellular source of IL-6 and OSM. To this end we isolated pancreatic macrophages and acinar cells from C57Bl/6-J mice. To mimic pancreatitis conditions in vitro we co-incubated pancreatic macrophages and freshly isolated CCK- (Cholecystokinin) stimulated acinar cells, which act as DAMPs. We could observe an upregulation of *Il6* mRNA as well as a significant IL-6 release from macrophages under in vitro pancreatitis conditions (Fig. 1c). Beside macrophages we could also observe a relevant upregulation of *Il6* mRNA in isolated acinar cells after stimulation with 1 µM CCK (Fig. 1d). In contrast to IL-6 we observed no upregulation of *Osm* mRNA (Fig. 1e) and only a moderate increase of OSM in macrophages on mRNA and protein level (Fig. 1f). Immunofluorescence co-labeling of IL-6 and CD68 provided further evidence that double-positive macrophages in the pancreas are a source of IL6 (Fig. 1g**)**.

### Blockage of IL-6 binding on CD4^+^ T cells mitigates their activation during AP

**Fig. 2:**
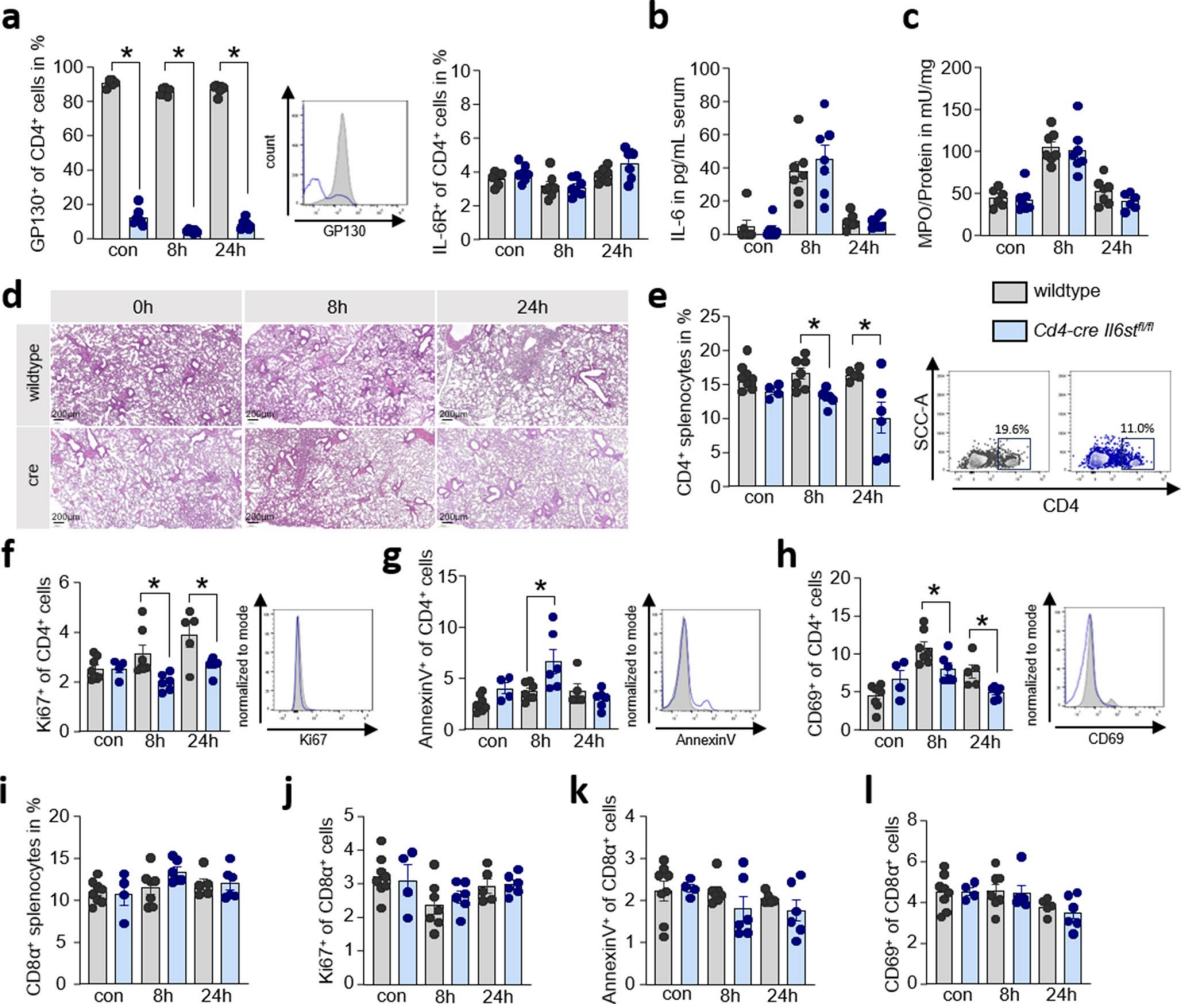
Blockage of IL-6 binding on CD4^+^ T cells mitigates their activation during AP*.* (**a**) Determination of GP130^+^ and IL-6R^+^ of CD4^+^ positive splenocytes was performed by flow cytometry analysis 8 h and 24 h after the onset of experimental pancreatitis in mice (con wildtype *n* = 6, other groups *n* = 7). (**b**) IL-6 serum concentrations were measured in caerulein-induced pancreatitis in *Cd4-cre Il6st*^*fl/fl*^ mice and wildtype littermates (con wildtype *n* = 6, other groups *n* = 7). (**c**) Analysis of Myeloperoxidase activity in lung tissue (con wildtype and 24 h *Cd4-cre Il6st*^*fl/fl*^*n* = 6, other groups *n* = 7) (**d**) and representative H&E staining of the lung (scale bar 200 μm). (**e**) Numbers of CD4^+^ splenocytes were analyzed by flow cytometry and CD4^+^ T cells were further characterized using Ki67^+^ (**f**), AnnexinV^+^ (**g**) and CD69^+^ (**h**) labeling by flow cytometry (con wildtype *n* = 8, con *Cd4-cre Il6st*^*fl/fl*^*n* = 4, 8 h wildtype *n* = 7, 8 h *Cd4-cre Il6st*^*fl/fl*^*n* = 6, 24 h wildtype *n* = 5, 24 h *Cd4-cre Il6st*^*fl/fl*^*n* = 6). Furthermore, CD8α^+^ splenocytes **(i)** were quantified by flow cytometry and in detail characterized by Ki67^+^ (**j**), AnnexinV^+^ (**k**) and CD69^+^ (**l**) labeling (con wildtype *n* = 8, con *Cd4-cre Il6st*^*fl/fl*^*n* = 4, 8 h wildtype *n* = 7, 8 h *Cd4-cre Il6st*^*fl/fl*^*n* = 6, 24 h wildtype *n* = 5, 24 h *Cd4-cre Il6st*^*fl/fl*^*n* = 6). All biological replicates (n) are shown with +/- SEM and means. Student’s t-test (two-tailed) (**a**,** e**,** f**,** g**,** h**) was used to determine statistical significance, which was indicated by an asterisk at a *p* value < 0.05

We aimed to address whether IL-6 or OSM have an impact on the significantly increased numbers of CD4^+^ T cells during the course of pancreatitis [19]. To investigate this, we induced AP by repetitive caerulein injections over eight hours in *Cd4-cre Il6st*^*fl/fl*^ mice (where Il6st is deleted in CD4^+^ T cells) and wildtype littermates (*Cd4-negativ Il6st*^*fl/fl*^). We confirmed the deletion of the GP130 expression on CD4^+^ T cells by flow cytometry analysis of splenic T cells, whereases no difference was observed in the IL-6 receptor’s expression (Fig. 2a). Next, we investigated the systemic inflammation by measurement of IL-6 concentrations in serum and myeloperoxidase activity in the lung tissue of mice. Wild type as well as mice lacking the GP130 receptor on CD4^+^ T cells showed a comparable systemic increase of IL-6 8 h after onset of pancreatitis (Fig. 2b). Similarly, the increase in myeloperoxidase activity in the lungs was the same in both groups (Fig. 2c). In H&E staining of lung sections, a comparable pancreatitis-induced lung damage was seen in *Cd4-cre Il6st*^*fl/fl*^ mice and wildtype littermates (Fig. 2d). The number of CD4^+^ T cells in spleen was significantly reduced in *Cd4-cre Il6st*^*fl/fl*^ mice 8 h and 24 h after onset of disease (Fig. 2e). At that time point we observed less Ki67^+^ CD4^+^ proliferating T cells (Fig. 2f) as well as more AnnexinV^+^ CD4^+^ apoptotic T cells (Fig. 2g). Furthermore, we labeled activated T cells with anti-CD69 antibodies. Interestingly, the interference of the IL-6 signaling on T cells reduced the number of activated CD69^+^ CD4^+^ T cells (Fig. 2h). CD8α^+^ T cell were unaffected (Fig. 2i). When we analyzed the expression of Ki67 (Fig. 2j), AnnexinV (Fig. 2k) and CD69 (Fig. 2l) on CD8α^+^ T cells we found no difference between *Cd4-cre Il6st*^*fl/fl*^ mice and controls. Taken together, these investigations indicated that IL-6 sustains T cell viability and enhances proliferation during caerulein induced AP.

### IL-6 promotes the proliferation of isolated CD4^+^ T cells

**Fig. 3:**
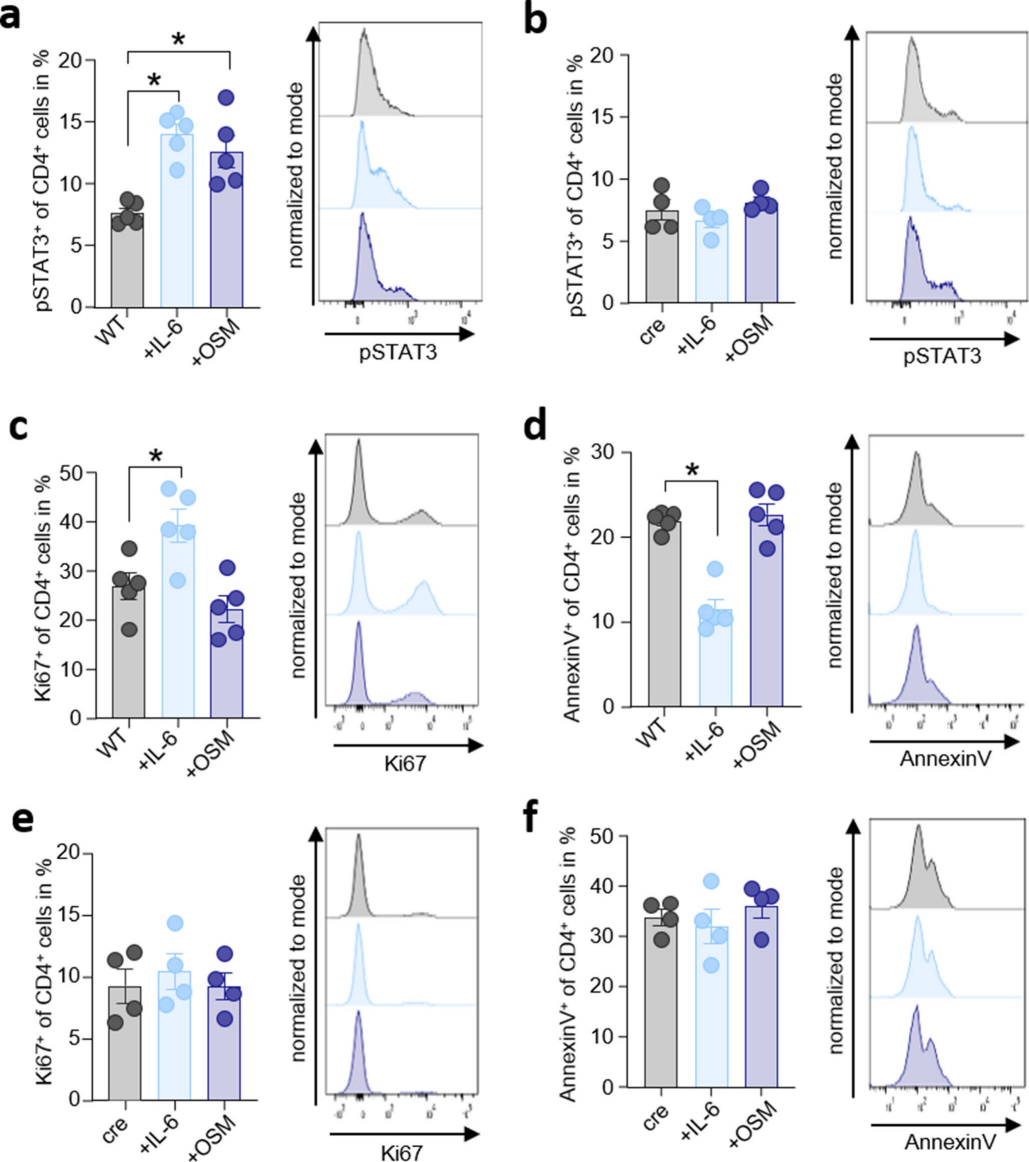
IL-6 promotes the proliferation of isolated CD4^+^ T cells*.* Isolated T cells from (**a**) wildtype mice (*n* = 5) and (**b**) *Cd4-Cre Il6st*^*fl/fl*^ mice (*n* = 4) were analyzed for pSTAT3^+^ cells by flow cytometry following exposure to 10 ng/mL IL-6 or 100 ng/mL OSM. T cells from untreated wildtype mice were further analyzed for Ki67^+^ (**a**) and AnnexinV^+^ cells (**b**) by flow cytometry and show a sensitivity for stimulation with IL-6, but not OSM. Blockage of IL-6 family member signaling in GP130 receptor knockout animals (cre) blocked the IL-6 effect on numbers of Ki67^+^ (**c**) and AnnexinV^+^ (**d**) T cells. All biological replicates (n) are shown with +/- SEM and means. 2-way ANOVA (**a**,** c**,** d**) was used to determine statistical significance, which was indicated by an asterisk at a *p* value < 0.05

In the next step, we investigated the effect of IL-6 and OSM on T cells in more detail. Therefore, we initially confirmed the efficacy of the IL-6 signaling blockade through the analysis of IL-6-stimulated CD4^+^ T cells from *Cd4-Cre Il6st*^*fl/fl*^ mice and wildtype littermates by flow cytometric analysis of pSTAT3. While IL-6 and OSM-stimulation results in the phosphorylation of STAT3 in CD4^+^ cells isolated from wildtype mice (Fig. 3a), T cells of *Cd4-Cre Il6st*^*fl/fl*^ mice did not show an increase of pSTAT3 (Fig. 3b). Isolated T cells from the spleen of wildtype mice showed increased proliferation (Ki67^+^ CD4^+^ T cells) after IL-6 treatment, but not after stimulation with OSM (Fig. 3c). We also analyzed via AnnexinV labeling if T cell apoptosis is mediated by IL-6 or OSM and observed that the stimulation with IL-6 reduces the number of AnnexinV^+^ CD4^+^ T cells significantly, while the treatment with OSM showed no effect (Fig. 3d). To verify the IL-6 specific influence on T cells we repeated this experiment with T cells isolated from the spleen of *Cd4-cre Il6st*^*fl/fl*^ mice. In the absence of GP130 on CD4^+^ T cells, the numbers of Ki67^+^ CD4^+^ and AnnexinV^+^ CD4^+^ T cells were unaffected after stimulation with IL-6 (Fig. 3e-f). Overall, our in vitro experiments supported our findings from the caerulein-induced AP mouse model, that IL-6 affects the systemic T cell response.

### IL-6 dependent T cell activation influences pancreatic damage during AP

**Fig. 4:**
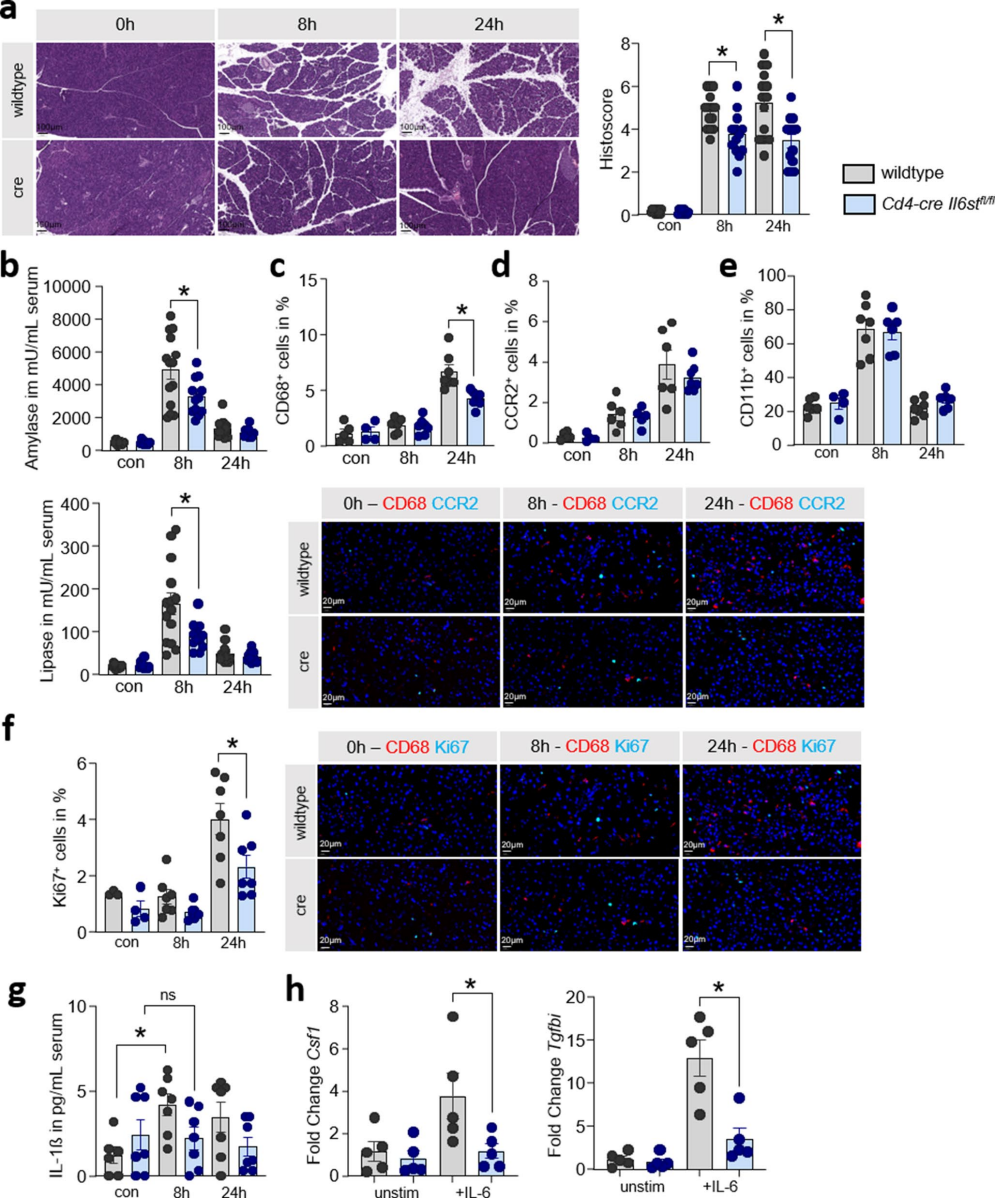
IL-6 dependent T cell activation influences the pancreatic damage during AP. Tissue damage was analyzed at 8 h and 24 h after the onset of AP in *Cd4-cre Il6st*^*fl/fl*^ transgenic and wildtype mice. (**a**) Representative H&E stained pancreas sections of wildtype and *Cd4-cre Il6st*^*fl/fl*^ mice (scale bar 100 μm). Histological scoring according to necrosis rate, leukoycte infiltration and edema development (*n* = 10–14). (**b**) Serum amylase and lipase measurements (*n* = 11–14). Quantitative evaluation of CD68^+^ cells (**c**) and CCR2^+^ cells (**d**) in immunofluorescence labeling of pancreatic tissue sections (*n* = 4–7, scale bar 20 μm). (**e**) Flow cytometry analyses of CD11b^+^ cell in blood samples of the mice (*n* = 4–7). (**f**) Quantitative evaluation of Ki67^+^ cells in pancreatic tissue section (*n* = 3–7). (**g**) IL-1ß serum concentrations were measured during caerulein-induced pancreatitis in *Cd4-cre Il6st*^*fl/fl*^ mice and wildtype littermates (*n* = 6–7). (**h**) Gene expression analysis of *Csf1* and *Tgfbi* in CD4^+^ T cells from wildtype and *Cd4-cre Il6st*^*fl/fl*^ mice following exposure to 10 ng/mL IL-6 (*n* = 5). All biological replicates (n) are shown with +/- SEM and means. Student’s t-test (two-tailed) was used to determine statistical significance, which was indicated by an asterisk at a *p* value < 0.05

Finally, we examined the effect of the IL6-dependent T cell activation on the pancreatic damage. H&E staining of pancreatic tissue sections from *Cd4-cre Il6st*^*fl/fl*^ transgenic and wildtype mice showed a reduced tissue damage at 8 h and 24 h after the onset of disease. Quantitative histological evaluation of necrosis, infiltration and edema in pancreatic sections revealed a significantly reduced pancreatic damage and supported the representative H&E figures (Fig. 4a). In line with the histological evaluation, serum amylase and lipase were significantly reduced in *Cd4-cre Il6st*^*fl/fl*^ mice (Fig. 4b). Macrophages are known to contribute to the disease severity. Therefore, we performed a detailed analysis of pancreatic macrophages by labeling CD68 in tissue sections of the inflamed pancreas. Quantification of immunofluorescence labeling experiments indicated significantly reduced numbers of CD68^+^ macrophages in *Cd4-cre Il6st*^*fl/fl*^ mice (Fig. 4c). Surprisingly, the infiltration was not affected by the reduced activation of T cells via IL-6, demonstrated by CCR2^+^ cells in the pancreas (Fig. 4d). The mobilization of monocytes and neutrophils during AP is independent of IL-6 mediated T cell activation, as we could show by flow cytometry analysis of CD11b^+^ cells in the blood of mice (Fig. 4e). Interestingly, the proliferation of pancreatic macrophages marked by Ki67 was reduced in the *Cd4-cre Il6st*^*fl/fl*^ mice (Fig. 4f). Serum cytokine levels of IL-1ß, which is predominantly produced by macrophages [6]are also influenced by a CD4^+^ specific IL-6 receptor knockout. The concentration of IL-1ß does not increase during pancreatitis in the *Cd4-cre Il6st*^*fl/fl*^ mice (Fig. 4g). These data indicated an influence of the IL-6 dependent systemic T cell activation on local macrophages. Gene expression analysis of IL-6 stimulated CD4^+^ T cells from *Cd4-cre Il6st*^*fl/fl*^ mice and wildtype littermates was performed by quantitative RT-PCR for growth factors *Csf1* and *Tgfbi*. We detected an IL-6 dependent upregulation of *Tgfbi* and *Csf1* mRNA in wildtype littermates, whereas the expression was unaffected in CD4^+^ T cells of *Cd4-Cre Il6st*^*fl/fl*^ mice (Fig. 4h).

## Discussion

In an acute episode of pancreatitis the initial damage of mainly pancreatic acinar cells results in a local inflammatory reaction, characterized by the activation of NFκB and the infiltration of neutrophiles and macrophages to the side of inflammation [8–11]. The infiltrating immune cells react on DAMPs by the release of various cytokines and chemokines which fuel the inflammatory cascade by the recruitment of additional immune cells and increase the local damage [6, 9]. An uncontrolled proinflammation induces a systemic inflammatory response syndrome (SIRS) which is associated with multiorgan dysfunction syndrome in the case of pancreatitis [23]. It is imperative that this potentially overwhelming reaction is balanced. In previous studies we were able to show that the pathomechanism of pancreatitis is characterized by the systemic activation of regulatory T cells, which counteract SIRS by the release of anti-inflammatory cytokines and suppress local and systemic inflammation. Especially in cases of severe acute pancreatitis increased serum levels of IL-10 could be observed, which indicates a shift from proinflammation to immunosuppression [6, 19, 24]. Our previous studies have demonstrated that T cell activation affects the course of pancreatitis [19, 24]. In the present study we examined in more detail the mechanism how the local pancreatic damage affects the systemic T cell activation during AP.

In serum samples of patients suffering from acute pancreatitis we observed a severity-dependent increase of IL-6 and OSM. Also, in our experimental animal model of AP induced by caerulein we could observe an increase of IL-6 serum concentrations. IL-6 is a pleiotropic cytokine and affects different processes like vascular diseases, lipid metabolism, insulin resistance, mitochondrial activities and neuropsychological mechanisms. Both pro- and anti-inflammatory properties are discussed for IL-6 [16, 25–28]. Previous studies have demonstrated that macrophages release significant amounts of IL-6. In our study we could verify a release of IL-6 and OSM from pancreatic macrophages under inflammatory conditions, such as pancreatitis. Besides macrophages we also identified acinar cells as source of IL-6. These data indicate that acinar cells have more immunoregulatory properties than previously assumed.

IL-6 family members mediate their effect locally via the IL-6 receptor, which is exclusively expressed on monocytes, lymphocytes and hepatocytes. Through alternative splicing and proteolytic separation of membrane-bound receptors, the soluble IL-6R (sIL-6R) can bind together with IL-6 to the ubiquitous GP130 receptor and thus exert its effect [29, 30]. Metalloproteases are responsible for this so-called IL-6 trans-signaling. Studies on the metalloprotease ADAM17 in caerulein-induced pancreatitis showed that an inhibition of ADAM17 reduces IL-6 trans-signaling and leukocyte recruitment into damaged pancreatic tissue [31]. Furthermore, it could be shown that IL-6 trans-signaling is responsible for pancreatitis associated lung damage in a mouse model of AP [32]. There have been many therapeutic approaches to block IL-6 signaling for the treatment of various inflammatory diseases and Tocilizumab was the first IL-6 blocking antibody, which was developed in the 1990 s [33]. The efficacy of tocilizumab for severity of pain and its impact on functioning in chronic pancreatitis are currently evaluated in a randomized, double-blind, placebo-controlled phase 2 trial [34]. The efficacy of these blocking antibodies is difficult to predict. For rheumatoid arthritis, tocilizumab is effective and well tolerated, but for other inflammatory diseases it may have a negative impact on disease progression, as clinical trials revealed unexpected outcomes. Among other problems, a compromised defense against pathogens and impaired tissue regeneration were observed [35–37]. In line with these observations, many studies have shown that a selective blockade of IL-6 trans-signaling via sgp130 seems more effective than a global blockade via an IL-6 neutralizing antibody [35]. Beside sepsis, myocardial infarction and bone fracture healing this has also been shown for caerulein-induced pancreatitis [32, 38, 39]. However, these studies did not actually reveal the effect of IL-6 in the pathomechanism of AP.

The prominent increase in serum IL-6 levels suggested that IL-6 significantly influences the systemic activation of the immune system. It is well described that T cells are activated during pancreatitis and play a role for the disease severity [6, 19]. Especially CD4^+^ T cells but not CD8α^+^ T cells have an impact on disease severity [40]. Previous studies have shown that IL-6 can act on T cells and protects them from apoptosis [41, 42]. Recent studies on acute pancreatitis have demonstrated that the number of peripheral CD4^+^ T cells in the blood is reduced in patients with severe disease [43, 44]. The occurrence of severe complications correlates directly with diminished numbers of circulating T cells [45]. Specifically, the infection of pancreatic necrosis, a major complication which is associated with severe form of pancreatitis [4]is linked to a T cell reduction in peripheral organs, such as the duodenum, where it results in an impaired intestinal barrier function [24]. While the reduction of T cells is primarily detected in severe cases, milder cases show no such significant impact [46]. A similar effect could be observed by using experimental mouse models of acute pancreatitis with different degrees of disease severity [24]. CD4^+^ T helper cells have the capacity to differentiate into diverse subclasses with distinct functions. During Pancreatitis increased levels of FOXP3^+^/CD25^+^ regulatory T cells (T_regs_) could be detected in mouse models [19] as well as in patients [47] in a severity dependent manner. T_regs_ act protective against hyperinflammation by suppressing T-effector cell activity, nevertheless an overriding T_reg_ activity could result in systemic immunosuppression, which may cause secondary infections, such as infected pancreatic necrosis [4]. A balanced suppression of T-effector cells acts protective in a mild form of the disease, while in severe cases T effector cells are needed to prevent the secondary infection of necrosis. Th17 cells are T-effector cells which act in a pro-inflammatory manner by secretion of IL-17A which drives macrophage and neutrophil activation [48, 49]. It is known that IL-6 enhances the proliferation of Th17 cells and also the induction of the transcription factor RORγt [20]. Our data show decreased T cell proliferation in the absence of GP130 on the surface of CD4^+^ T cells, which results in a reduced pro-inflammatory response of macrophages in the pancreas. The depletion of T cells or a diminished activation by IL-6 attenuate the disease severity in a mild model of acute pancreatitis and suggest a critical role of T cells in the immune regulation during acute pancreatitis. Further experiments are need to address the IL-6 mediated T cell differentiation and to decipher the role of T cell subclasses during acute pancreatitis.

Surprisingly, we could not observe these effects for OSM, another member of the IL-6 family. To test whether this activation functions exclusively via the GP130 receptor, we used a CD4^+^ T cell specific knock out of the GP130 receptor. Indeed, our results showed an IL-6-dependent activation of proliferation and inhibition of apoptosis via the GP130 receptor. Moreover, in a caerulein-induced mouse model of AP we observed an impaired T cell activation in *Cd4-cre Il6st*^*fl/fl*^ mice. Importantly, this does not curtail the relevance for IL-6 serum levels or lung damage. Taken together, these results demonstrate a relevant role of IL-6 on isolated T cells and in particular in experimental AP. Reduced T cell activation leads to a significant reduction of pancreatic damage, and severity markers such as serum amylase and lipase indicate a mitigation of the disease severity. This effect is mediated by reduced numbers of CD68^+^ macrophages within the pancreas. Interestingly, not the migration into the organ is influenced but the proliferation of leukocytes. Our data demonstrated that the IL-6-mediated activated of T cells is accompanied by an increased mRNA expression of *Tgfbi* and *Csf1*. Both growth factors could influencing the local macrophage proliferation. It is assumed that the reduced T cell activation in *Cd4-cre Il6st*^*fl/fl*^ mice mitigates macrophage proliferation and therefore the local pancreatic damage.

A limitation of the present study is, that the authors clearly demonstrate IL-6-mediated T cell activation in animal experiments, no such patient data were collected. While patient samples demonstrated a decline in T cell numbers in accordance with severity, the mechanisms of activation and differentiation remains to be elucidated [43–45]. Concurrently, a positive correlation between serum IL-6 levels and severity was observed. A further limitation of our study is that caerulein-induced pancreatitis represents a mild model of the disease. At this point, it would be interesting to also investigate IL-6-mediated T cell activation in a severe model of acute pancreatitis and evaluate whether similar effects can be observed. In future studies, in addition to the activation of T cells, the differentiation of the cells should also be investigated in more detail in order to gain deeper insights into the T cell-macrophage crosstalk.

In conclusion, our study revealed a critical role of IL-6 dependent T cell activation and its influence back on the local macrophage proliferation. This study verified that the T cell activation of AP is mediated by IL-6. Our results suggest that IL-6 significantly regulates the balance between inflammation and immunosuppression during pancreatitis and links the local and adaptive immune reactions. Furthermore, our findings show an influence of the systemic immune response on pancreatic damage and disease severity. Apparently, the initial local tissue damage triggers systemic immune reaction and the strength of the systemic immune response provokes macrophage proliferation and therefore influences severity of the pancreatic damage.

## Materials and methods

### Ethics Declaration

All animal experiments were reviewed and approved by the local animal care committee (Landesamt für Landwirtschaft, Lebensmittelsicherheit und Fischerei Mecklenburg-Vorpommern, protocol number: 7221.3-1-036/23) and were performed in accordance with German Animal Protection Law.

All human serum samples were collected between 2005 and 2020 at the University Medicine Greifswald (UMG). The studies are approved by the local ethical committee of the UMG (protocol number: III UV 91/03). Written informed consent was given from blood donors as well as patients.

### Animal Model

All mice were maintained at the central animal facility of the UMG under specific pathogen free conditions in ventilated cages with ad libitum food and water as well as with controlled environmental conditions (temperature, humidity) and a 12 h/12 h light/dark cycle. All animals had not previously been used in other experiments and were untreated until the time of pancreatitis induction. For all experimental setups, mice were matched for age and sex.

C57Bl/6-J mice were obtained from Charles River (Sulzfeld, Germany). *Cd4-cre Il6st*^*fl/fl*^ mice on a C57BL/6-J background were generated from *Il6st*^fl/fl^ mice (*Il6st*^*tm1Wme*^*Tg(Dmp1-cre)1Jqfe*) by crossing with *Cd4-cre*^pos^ mice (*Tg(Cd4-cre)1Cwi/BfluJ*), both obtained from the central animal facility of the UMG.

8- to 16-week-old *Cd4-cre Il6st*^*fl/fl*^ mice were used for the caerulein induced AP model, as previously described [9, 11]. Littermates were taken as wildtype control in experimental groups. AP was induced by hourly repetitive caerulein i.p. injection (50 µg/kg/bodyweight) (4030451, Bachem) over 8 h. Mice were euthanized by cervical dislocation at the experimental end point (8–24 h). Subsequently, pancreas, lung and spleen were harvested for further analysis.

### Antibodies and Reagents

The following antibodies were used for immunofluorescence labeling and flow cytometry: Anti-IL-6 antibody (dilution 1:200, ab208113, abcam), anti-CD68 rat monoclonal antibody (dilution 1:200, SM155OP, Origene), anti-CCR2 antibody (dilution 1:200, ab273050, abcam), anti-Ki-67 (dilution 1:200, IHC-00375, Bethyl), AlexaFluor647 donkey anti-rabbit (dilution 1:200, A31573, Invitrogen), anti-rat-Cy3 (dilution 1:200, 112–165-062, Jackson ImmunoResearch), anti-CD4-Brilliant-Violet650 (dilution 1:50, 100546, BioLegend), anti-AnnexinV-FITC (dilution 1:10, 640945, BioLegend), anti-Ki67-APC (2 µL/test, 130-119-357, Miltenyi Biotec), anti-CD130 (gp130)-PE (dilution 1:50, 149404, BioLegend), anti-CD69-Brilliant Violet510 (dilution 1:50, 310936, BioLegend), anti-CD8α-PE (dilution 1:50, 100708, BioLegend), anti-STAT3 Phospho (dilution 1:10, 651004, BioLegend), anti-CD126(IL-6Rα)-PE/Cyanine7 (dilution 1:50, 115814, BioLegend).

Recombinant mouse IL-2 protein was obtained from Miltenyi Biotec (130-120-133). Recombinant mouse IL-6 protein (406-ML) and recombinant mouse Oncostatin M protein (495-MO) were purchased from R&D Systems. Recombinant mouse M-CSF was obtained from BioLegend (576406).

### Isolation of Macrophages from Pancreatic Tissue

Pancreatic tissue from untreated C57BL/6-J mice was dissociated using the *Multi Tissue Dissociation Kit 3* (130-110-204, Miltenyi). Therefore, the pancreas was incubated under shaking at 37 °C for 10 min in enzyme-buffer mix. After adding RPMI medium containing 10% fetal calf serum and 1% Penicillin/Streptomycin the tissue was homogenized with the *gentleMACS dissociator* (130-093-235, Miltenyi) at 52 s for 287 runs. The cell suspension was passed through a 40 μm cell strainer to remove acinar cells and then cultured in medium containing 20 ng/mL macrophage colony-stimulating factor (M-CSF) over 7–10 days to induce the proliferation of macrophages. Macrophages were co-incubated with freshly isolated acinar cells for 24 h to mimic in vitro pancreatitis conditions. For the purpose of further analysis, the stimulation media was removed and the macrophages were washed twice with Phosphate Buffered Saline (PBS) to remove residual acinar cells.

### Isolation of Pancreatic Acinar Cells

Acinar cells were isolated by collagen digestion as previously described [9, 11]. In the presence of 1 mg collagenase of Clostridium histolyticum (EC.3.4.24.3) from Serva (lot no. 14007, Heidelberg, Germany) the pancreas was dissociated in Dulbecco’s modified Eagle medium containing 2% bovine serum albumin, 1% Penicillin/Streptomycin and 10 mM HEPES at 37 °C for 30 min under shaking. The suspension was then passed through a strainer, centrifuged to remove debris and collagenase solution and then stimulated with 1 µM cholecystokinin (CCK, C2901-1MG, Sigma Aldrich) for 1 h, 2 h, 4 h and 6 h.

### Cell Isolation for Flow Cytometry

Splenocytes for flow cytometry analysis were isolated from *Cd4-cre Il6st*^*fl/fl*^ mice and wildtype littermates. Therefore, the spleen was pressed through a 70 μm cell strainer. This single cell suspension was then treated with a 1x lysis buffer (10x buffer: 1.5 M NH4Cl, 0.1 M KHCO3, 10 mM EDTA ∙ 2Na) for 5 min at room temperature to remove erythrocytes. The reaction was stopped with PBS, washed by centrifugation and the number of living cells was determined by *CASY Cell Counter* (OMNI Life Science). All centrifugations were performed at 300 g for 6 min at 4 °C. CD4^+^ T cells were isolated from this single-cell suspension using the *CD4*^*+*^*T Cell Isolation Kit* (130-104-454, Miltenyi). T cells were cultured in *TexMACS™* Medium (130-097-196, Miltenyi) with IL-2 (10 ng/mL). Depending on the stimulation conditions, the cells were treated with IL-6 (10 ng/mL) or OSM (100 ng/mL) for 24 h.

For the characterization of the cells by flow cytometry, the cells were transferred to a FACS-tube. All dilutions were performed in staining buffer (PBS, 2% FCS, 0,02% sodium azide, 2 mM EDTA). First, the cells were pre-incubated with *FcR Blocking Reagent* (130-092-575, MiltenyiBiotec) to block unspecific binding of antibodies to the Fc-receptor. Next, antibodies to extracellular markers were used to label CD4^+^, CD8α^+^, AnnexinV^+^, CD69^+^, IL-6Rα^+^ and GP130^+^ cells, and diluted 1:50 in staining buffer for 30 min at 4 °C. After permeabilization of cell membrane and nucleus (Transcription Factor Staining Buffer Set, 130-122-981, Miltenyi Biotec) the cell suspensions were again incubated with FcR Blocking Reagent and treated with antibody to mark Ki67^+^ and pSTAT3^+^ cells. Finally, the expression of activation marker as well as proliferation marker were analyzed by flow cytometry (BD, *LSRII*) and calculated by *FlowJo*.

### Myeloperoxidase Measurement

Lung samples were homogenized in 20 mM potassium phosphate buffer (pH 7.4) on ice with a dounce homogenizer. After centrifugation the cell pellet was dissociated in extraction buffer (50 mM potassium phosphate buffer (pH 6.0), 0.5% cetyltrimethylammoniumbromide) by four freeze-thaw cycles and additional sonification. The myeloperoxidase activity was measured in 50 mM potassium phosphate buffer (pH 6) supplemented with 0.53 mM O-dianisidine and 0.15 mM H_2_O_2_ at 30 °C for 10 min in a Spectrophotometer (Spectramax, Molecular Devices, San Jose, CA USA). The enzyme activity was determined against a standard (Cat# 475911, Calbiochem) and adjusted to the protein content of the lung sample [9].

### Serum Amylase and Lipase Measurement

Amylase and lipase activities in the serum were determined using appropriate colorimetric kits from Roche (AMYL2 Ref: 03183742122; LIPC Ref: 03029590322) according to the manufacturers protocol.

### Cytokine Measurement

IL-6 concentration was measured in cell culture supernatant of stimulated macrophages and acinar cells which were seeded in a 96-Well plate by *Mouse T Helper Cytokine Panel* (741044, BioLegend).

The serum cytokine levels of IL-6 in human samples were measured by *Human Cytokine Panel 2* (741378, BioLegend) and in murine samples by *Cytometric Bead Array (CBA) Mouse inflammation kit* (552364, BD Bioscience). Serum IL-1β concentrations were measured by *Legendplex – Custom Mouse Panel 744* (900001844, BioLegend).

OSM levels were evaluated in serum and cell culture supernatant by *Mouse Oncostatin M Immunoassay* (MSM00, R&D Biotech). For human serum samples the *Human Oncostatin M (OSM) DuoSet ELISA Kit* was used (DY295, R&D Biotech).

### Histology

Sections of pancreas and lung tissue were fixed in 4.5% buffered formaldehyde solution > 24 h. Paraffin-embedded tissue samples were cut in 2 μm slides and stained for H&E. Cryo-embedded pancreas samples in TissueTec were cut in 2 μm slides. Immunofluorescence labeling of CD68, IL-6, CCR2 and Ki67 (dilution 1:200, overnight) was performed. Secondary antibodies were used in a 1:200 dilution and incubated for 1 h at room temperature. Slides were scanned on a *Pannoramic MIDI II* (Sysmex) platform.

### RNA Extraction and RT–qPCR

Total RNA was extracted from macrophages and in addition from freshly isolated acinar cells in a 6-well plate in TRIzol reagent (155966018, Invitrogen™) according to the manufacturer’s instructions and reverse-transcribed into cDNA using 2 µg RNA; 5 µM OligodT primers; 0.2 µg random primers; 0.5 µM dNTP Mix; 1× First Strand Buffer (18080044, Invitrogen); 10 µM DTT; 40 Units RNasin Ribonuclease Inhibitor (N251B, Promega) and 200 Units M-MLV RT (28025013, invitrogen). Quantitative PCR (RT-qPCR) using SYBR-green was used to analyze the expression of genes of interest. The cDNA samples (1:10 diluted) were amplified by real-time PCR with 1× SYBR Green PCR Master Mix (4367659, applied biosystems) and 300 ng gene-specific oligonucleotide primers (reverse and forward) in three technical replicates. The relative mRNA levels were normalized to *Rn5s* and to the relative expression in untreated samples.

The following oligonucleotide primers were used: *Rn5s* forward 5′-GCCCGATCTCGTCTGATCTC-3′ reverse 5′-GCCTACAGCACCCGGTATTC-3′, *Il6* forward 5′- CCAGAGTCCTTCAGAGAGATACA-3′ reverse 5′-CCTTCTGTGACTCCAGCTTATC-3′, Osm forward 5′-ATGCAGACACGGCTTCTAAGA-3′ reverse 5′-TTGGAGCAGCCACGATTGG-3′, *Csf1* forward 5′-GCCTCCTGTTCTACAAGTGGAAG-3′ reverse 5′-ACTGGCAGTTCCACCTGTCTGT-3′, Tgfbi forward 5′-TCTTCAAACAGGCGTCAGCG-3′ reverse 5′-AAACTGAGAGAAACTGGCG-3′.

### Statistical Analysis and Data Plotting

All statistical tests and graphical representations were carried out in *GraphPad Prism* (version 10.0). In all graphs SEM and means are indicated. Normality was analyzed using Shapiro-Wilk normality test. Unpaired Student’s t test (two-tailed) or one-way analysis of variance (ANOVA) was performed on normally distributed samples, and Mann-Whitney or Kruskal-Wallis tests were calculated on samples that did not pass the normality test. Significant differences with a *p* value < 0.05 were marked by an asterisk. Flow cytometric plots are illustrated by *FlowJo* (version 10.0) and are representative of experimental groups. *PowerPoint* (Office 2019) was used to modify and assemble plots for presentation.

## Data Availability

No datasets were generated or analysed during the current study.
